# Regulation of Peroxisome Proliferator-Activated Receptors by E6-Associated Protein

**DOI:** 10.1155/2008/746935

**Published:** 2008-12-18

**Authors:** Lakshmi Gopinathan, Daniel B. Hannon, Russell W. Smith III, Jeffrey M. Peters, John P. Vanden Heuvel

**Affiliations:** ^1^The Huck Institutes of the Life Sciences, The Pennsylvania State University, University Park, PA 16802, USA; ^2^Center for Molecular Toxicology and Carcinogenesis, Department of Veterinary & Biomedical Sciences, The Pennsylvania State University, University Park, PA 16802, USA

## Abstract

Peroxisome proliferator-activated receptors (PPARs) are nuclear receptors (NRs) that regulate genes involved in lipid and glucose metabolism. PPAR activity is regulated by interactions with cofactors and of interest are cofactors with ubiquitin ligase activity. The E6-associated protein (E6-AP) is an E3 ubiquitin ligase that affects the activity of other NRs, although its effects on PPARs have not been examined. E6-AP inhibited the ligand-independent transcriptional activity of PPAR*α* and PPAR*β*, with marginal effects on PPAR*γ*, and decreased basal mRNA levels of PPAR*α* target genes. Inhibition of PPAR*α* activity required the ubiquitin ligase function of E6-AP, but occurred in a proteasome-independent manner. PPAR*α* interacted with E6-AP, and in mice treated with PPAR*α* agonist clofibrate, mRNA and protein levels of E6-AP were increased in wildtype, but not in PPAR*α* null mice, indicating a PPAR*α*-dependent regulation. These studies suggest coordinate regulation of E6-AP and PPAR*α*, and contribute to our understanding of the role of PPARs in cellular metabolism.

## 1. INTRODUCTION

The peroxisome proliferator activated receptors (PPARs) are nuclear hormone receptors that regulate lipid and glucose metabolism, and are critical to the maintenance of cellular energy homeostasis. In addition, they regulate several biological processes such as inflammation, differentiation, apoptosis, and wound healing [1, 2]. Three different subtypes of PPARs mediate these responses: PPAR*α*, PPAR*β*, and PPAR*γ*. PPAR*α* is activated by fatty acids, fatty acid metabolites, and peroxisome proliferators, a diverse group of xenobiotics that includes the fibrate hypolidemic drugs, phthalate esters, and herbicides [3]. Regulation of gene expression by PPAR*α* follows the classical ligand-dependent transcription factor mechanism. Upon ligand binding, PPAR*α* binds to PPAR-response elements (PPREs) in the promoter of target genes as a heterodimer with retinoid X receptor (RXR). The multiple protein-PPAR*α* interactions that occur in the transcription complex are important for proper target gene regulation [4]. These proteins, often called coregulators, can increase (coactivators) or repress (corepressors) transcriptional activity. Some coregulators possess enzymatic activity such as histone acetyl transferase or histone deacetylase, and modulate chromatin structure to regulate gene transcription [5]. Several proteins with ubiquitin ligase activity have been characterized in the last few years as coregulators for nuclear receptors. The recruitment of ubiquitin-proteasome components to the promoters of nuclear receptor target genes suggest an additional layer of transcriptional regulation by the ubiquitin-proteasome pathway [6–8].

This study examines regulation of PPAR*α* by E6-associated protein (E6-AP), a protein linked to the Angelman syndrome and an E3 ubiquitin ligase that belongs to the HECT (homologous to the E6-AP C-terminus) family [9]. Ablation of E6-AP in mice is associated with steroid hormone resistance and reproductive defects [10]. E6-AP coactivates nuclear receptors such as the estrogen receptor (ER) and the progesterone receptor [11]. In addition, it mediates proteasomal degradation of proteins such as the nuclear receptor coactivator AIB1 [12], and tumor suppressors Rb (retinoblastoma protein), and p53 [13–15]. The studies presented here suggest a role for the ubiquitin ligase function of E6-AP in regulating PPAR*α* activity.

## 2. MATERIALS AND METHODS

### 2.1. Plasmids

The plasmids pBKRSV-E6AP, pBKRSV-E6AP-C833S, and pM-E6AP were a kind gift from Dr. Zafar Nawaz (Department of Cell Biology, Baylor College of Medicine, Houston, Tex, USA). The construction of the pVP16-PPAR*α* plasmid has been described previously [16]. The pFR-luciferase (UAS luciferase) plasmid was purchased from BD Biosciences Clontech (Palo Alto, Calif, USA), while pRL/TK and pRL/CMV were from Promega (Madison, Wis, USA). The peroxisome proliferator response element (PPRE) reporter pACO (-581/-471) G.Luc was supplied by Dr. Jonathan Tugwood (AstraZeneca Maccelsfield, UK) and has been described previously [17]. The pcDNA3.1/V5-His-PPAR*α* plasmid has been described previously [16]. The pcDNA3.1/FLAG-PPAR*β* and pcDNA3.1-PPAR*γ* plasmids were a kind gift from Dr. Curtis Omiecinski (Department of Veterinary and Biomedical Sciences, The Pennsylvania State University, Pa, USA).

### 2.2. Transfections and reporter assays

FaO cells (maintained in DMEM/Nutrient F-12 Ham with 8% serum and 100 units each of penicillin and streptomycin) were transfected using Lipofectamine RNAiMAX reagent (Invitrogen, Carlsbad, Calif, USA), following manufacturer's instructions. Lipofectamine (Invitrogen) was used to transfect 293T cells (maintained in HG-DMEM with 8% serum and 100 units each of penicillin and streptomycin) according to the manufacturer's instructions. For reporter assays examining transient PPRE activity, all transfections included pRL/CMV (Promega) to control for transfection efficiency and ACO-luciferase. When indicated, following transfection, cells were treated with 0.1% DMSO or 5 *μ*M MG132 for 6 hours. For reporter assays examining transient Gal4 response element activity, all transfections included pRL/CMV to control for transfection efficiency and pFR-Luciferase. In Gal4 response element assays, cells were treated for 6 hours with 0.1% DMSO or 50 *μ*M Wy-14,643 before lysis. Cells were lysed and *renilla* and *firefly* luciferase activities were examined using the Dual Luciferase Assay kit (Promega). Luciferase activity was corrected for transfection efficiency (pRLTK/pRLCMV) and extraction yield (via total protein assay).

### 2.3. Real-time PCR

Total RNA was isolated using Tri Reagent (Sigma, St. Louis, Mo, USA) according to the manufacturer's protocol. The total RNA was reverse transcribed using the ABI High Capacity cDNA Archive Kit (Applied Biosystems, Foster City, Calif, USA). Standard curves were made using serial dilutions from pooled cDNA samples. Real Time PCR was performed using the SYBR Green PCR Mater Mix (Applied Biosystems) according to the manufacturer's protocol and amplified on the ABI Prism 7300 Sequence Detection system. Messenger RNA levels of all genes were normalized to *β*-actin mRNA. Primer sequences (5′-3′) are E6-AP forward: gaaatgaggcctgcacgaat, E6-AP reverse: gaagaaaagttggacaggaagca, *β*-actin forward: ggctctatcctggcctcactg, *β*-actin reverse: cttgctgatccacatctgctg. Primers for Acyl CoA Oxidase (ACO) Cytochrome P450 IV A10 (CYP4A10), Angiopoietin-like protein 4 (Angplt4), have been described previously [16]. Sequences (5′-3′) for other genes measured are fatty acid binding protein 1 (FABP1) forward: ttctccggcaagtaccaagtg, FABP1 reverse: tcatgaagggctcaaagttctctt, Enoyl CoA Hydratase forward: cccgcaggatctttaacaagc, Enoyl CoA Hydratase reverse: cactgtccatgttgggcaag.

### 2.4. Western blotting

Mouse livers were homogenized in lysis buffer containing 50 mM Tris (pH 8), 120 mM NaCl, 0.5% Nonidet P-40, and 1 : 100 dilution of protease-inhibitor cocktail (Sigma) after which particulates were removed by centrifugation. Liver lysates were subjected to SDS/PAGE. Proteins were transferred to Immobilon-PVDF membrane (Millipore), followed by western using anti-E6AP antibody (H-182, Santa Cruz Biotechnology). Band intensities were quantitated using Optiquant Acquisition and Analysis Software.

### 2.5. Mice

8-week old-male wild-type, and PPAR*α*-null mice [18] were housed in a light (12 hours light/12 hours dark) and temperature (25°C) controlled environment in microisolator cages. Mice were gavaged daily with either vehicle control (corn oil) or 500 mg clofibrate/kg body weight for 14 days. Mice were euthanized, livers weighed and homogenized, RNA or protein isolated for analysis as described above.

## 3. RESULTS

### 3.1. E6-AP inhibits the transcriptional activity of PPAR*α*, PPAR*β*, and PPAR*γ*

**Figure 1:**
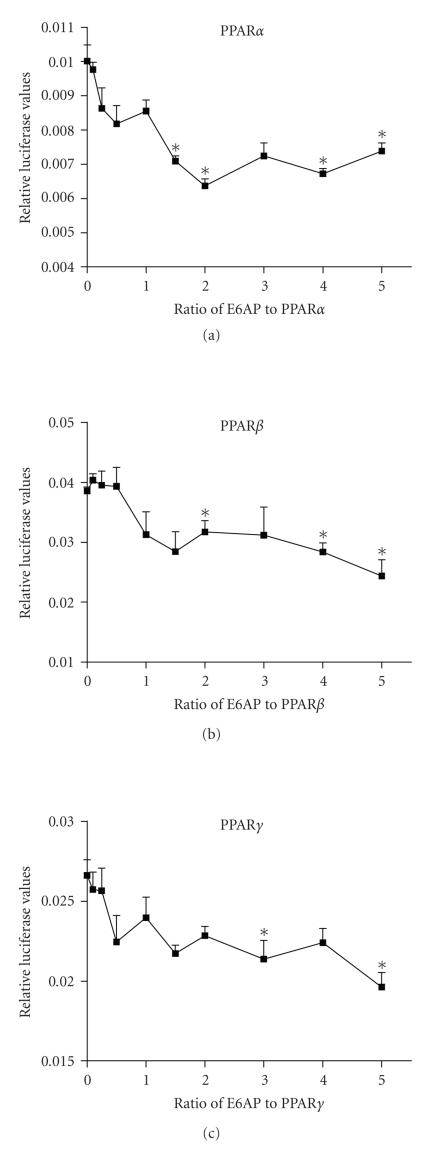
E6-AP inhibits the transcriptional activity of PPAR*α*, PPAR*β*, and PPAR*γ*. 293T cells were transfected with plasmids expressing 4X-ACO-Luciferase, pRLCMV, PPAR*α*, PPAR*β* or PPAR*γ*, and E6-AP. Cells were lysed and luciferase activity was corrected for transfection efficiency and protein. Asterisks indicate a significant difference in luciferase values when compared to the 0 ratio group. (*P < .05 with statistical analysis using ANOVA). The graphs are representative of 3 independent experiments.

The transcriptional activity of PPAR*α*, PPAR*β*/*δ*, and PPAR*γ* isotypes was examined in the presence of E6-AP, by measuring the activity of a reporter gene under the control of a natural PPRE. As seen in Figure 1, transfecting increasing amounts of E6-AP inhibited PPAR transactivation in a dose-dependent manner for all three PPAR isotypes. A 40% decrease in transactivation was observed for PPAR*α* and PPAR*β*, with a statistically significant decrease observed first at a ratio of 1.5 : 1 for E6-AP : PPAR*α*, and 2 : 1 for E6-AP : PPAR*β*. A 30% decrease in transactivation was seen with PPAR*γ*, with a statistically significant decrease observed first at a ratio of 3 : 1 for E6-AP : PPAR*γ*. No changes were observed in ligand-induced activity of the receptors in the presence of E6-AP (data not shown).

### 3.2. E6-AP overexpression affects PPAR*α* target genes

**Figure 2:**
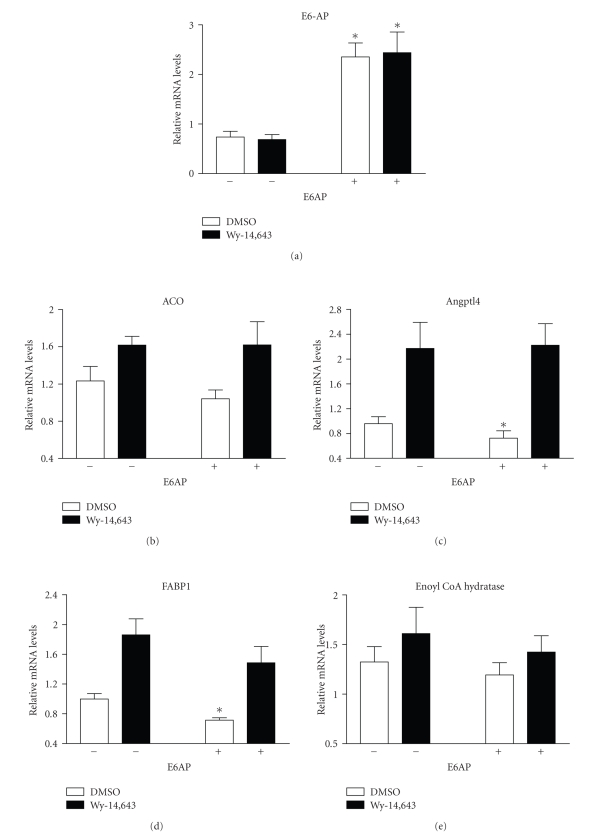
E6-AP expression results in decreased mRNA levels of PPAR*α* target genes. FaO cells were transfected with empty vector or plasmid expressing E6-AP, followed by treatment with 0.1% DMSO or 50 *μ*M Wy-14,643 for 6 hours. Total RNA was isolated from the cells and real-time PCR was performed on reverse transcribed RNA. Asterisks indicate a significant difference when compared to the corresponding control group. (*P < .05 with statistical analysis using ANOVA). The graphs represent mean values obtained from 2 independent experiments.

To further examine the effect of E6-AP on the transcriptional activity of PPAR*α*, E6-AP was expressed in FaO cells by transient transfection, followed by treatment with PPAR*α* ligand Wy-14,643. The effect of E6-AP overexpression on mRNA levels of endogenous PPAR*α* target genes was measured. The genes examined (angiopoietin-like protein 4 or Angplt4, fatty acid binding protein 1 or FABP1, acyl CoA oxidase or ACO and enoyl CoA hydratase) were chosen based on their role in PPAR*α*-mediated lipid metabolism, or were previously identified in gene expression microarrays in FaO cells [19]. As seen in Figure 2, E6-AP expression resulted in statistically significant changes in mRNA levels Angplt4 (24% decrease) and FABP1 (28% decrease), in the absence of ligand. As previously seen with PPRE-driven reporter assays (Figure 1), no changes were seen in ligand-induced mRNA levels of PPAR*α* target genes with E6AP expression.

### 3.3. E6-AP interacts with PPAR*α*

**Figure 3:**
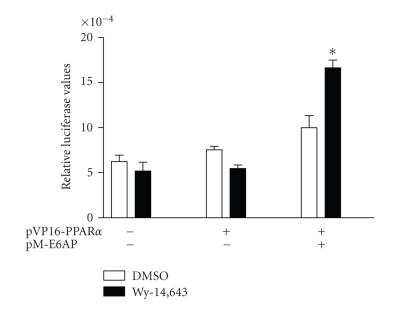
E6-AP interacts with PPAR*α*. 293T cells were transfected with plasmids expressing pFR-Luciferase, pRLCMV, pVP16-PPAR*α*, pM-E6-AP. Cells were lysed and luciferase activity was corrected for transfection efficiency and protein. Asterisks indicate a significant difference in Wy-14,643 induction when compared to the corresponding DMSO group. (*P < .05 with statistical analysis using ANOVA). The graph is representative of 2 independent experiments.

To examine if the effect of E6-AP on the transcriptional activity of PPAR*α* was due to a direct interaction between the two proteins, mammalian-two-hybrid assays were performed using plasmids expressing PPAR*α* fused to the pVP16 activation domain and E6-AP in the pM vector. The Gal4 response element reporter (pFR-luciferase) was used to assess the interaction between E6-AP and PPAR*α*. As seen in Figure 3, induction with PPAR*α* agonist Wy-14,643 was seen only when E6-AP was coexpressed with PPAR*α*, indicating an interaction between the two proteins.

### 3.4. The E3 ubiquitin ligase function of E6-AP is required for inhibition of PPAR*α* transcriptional activity

**Figure 4:**
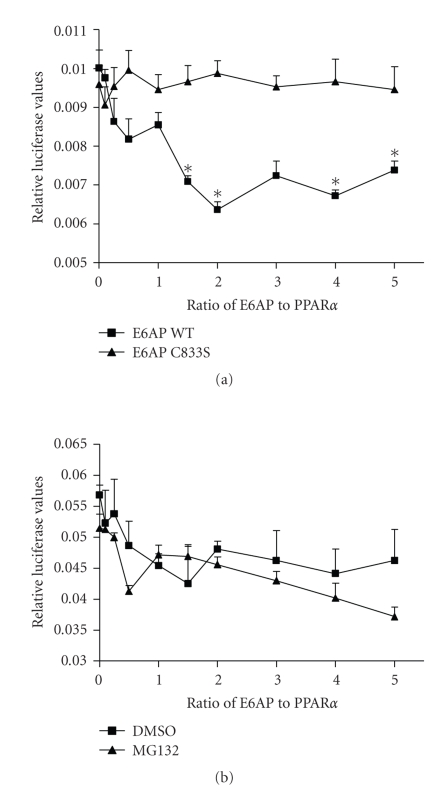
E3 ubiquitin ligase activity of E6-AP is required for regulating PPAR*α* transactivation. (a) 293T cells were transfected with plasmids expressing 4X-ACO-Luciferase, pRLCMV, PPAR*α*, E6-AP, or E6-AP-C833S that is defective in ubiquitin ligase function. (b) Cells were treated with 0.1% DMSO or 5 *μ*M MG132 for 6 hours. Cells were lysed and luciferase activity was corrected for transfection efficiency and protein. Asterisks indicate a significant difference when compared to the corresponding 0 ratio group. (*P < .05 with statistical analysis using ANOVA). The graph is representative of 3 independent experiments.

In order to determine if the effect of E6-AP on PPAR*α* activity was mediated by the E3 ubiquitin ligase function of E6-AP, E6-AP C833S, a mutant defective in ubiquitin ligase function was used. Unlike the changes in reporter activity seen with wildtype (WT) E6-AP (Figure 1), transfecting increasing amounts of E6-AP-C833S did not result in any changes in activity (Figure 4(a)), indicating that the ubiquitin ligase function of E6-AP is required for regulating the transcriptional activity of PPAR*α*. These differences were not due to different transfection efficiencies, since both E6-AP WT and E6-AP-C833S expressed equally well in these cells (data not shown). To further assess if E6-AP-mediated inhibition of PPAR*α* transactivation was via proteasomal degradation, PPRE-dependent reporter assay was performed in the presence of proteasome inhibitor MG132. Transfecting increasing amounts of E6-AP resulted in decreased reporter activity in the presence and absence of MG132 (Figure 4(b)), indicating that E6-AP-mediated inhibition of PPAR*α* transactivation occurs via a proteasome-independent mechanism.

### 3.5. E6-AP is regulated in vivo in a PPAR*α*-dependent manner

**Figure 5:**
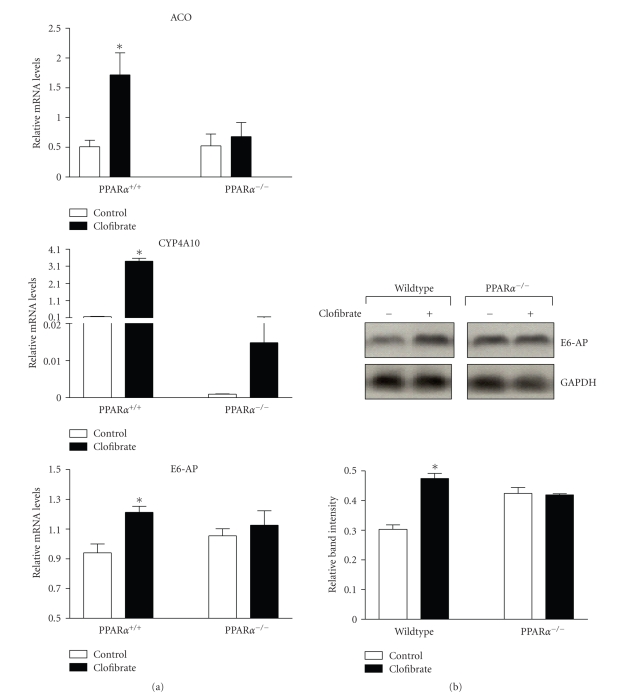
E6-AP is induced by clofibrate in a PPAR*α*-dependent manner. Wildtype and PPAR*α* null mice were treated with control vehicle or clofibrate for 2 weeks. Groups of five mice were used for each treatment. (a) Total RNA was isolated from liver and mRNA levels were measured using real-time PCR. (b) Protein isolated from liver was analyzed for E6AP expression by western blot. The graph (lower panel) depicts mean (*n* = 5) band intensity. Asterisks indicate a significant increase in clofibrate induction when compared to the PPAR*α* null group. (*P < .05 with statistical analysis using ANOVA).

Since NR-mediated transcriptional regulation of E3 ligases has been demonstrated in a few studies [20–22], regulation of E6-AP in response to PPAR*α* ligand was examined in vivo. Wild type and PPAR*α* null mice were maintained on a clofibrate or control diet for two weeks, following which their livers were analyzed for mRNA and protein levels of E6-AP. As expected, mRNA levels of known PPAR*α* target genes (acyl CoA oxidase or ACO and cytochrome P450 IV A10 or CYP4A10) were induced in response to clofibrate and this response was defective in PPAR*α* null mice (Figure 5(a)). E6-AP mRNA (Figure 5(a)) and protein (Figure 5(b)) levels were significantly increased in wildtype mice in response to clofibrate, but not in PPAR*α* null mice, indicating a PPAR*α*-dependent regulation.

## 4. DISCUSSION

The regulation of PPARs by the ubiquitination has been the subject of limited investigation. However, recent studies suggest ligand-mediated regulation of PPARs via the ubiquitin-proteasome system, although no ubiquitin ligase has been identified. PPAR*α* and PPAR*β* ligands affect receptor ubiquitination and protein levels [23–26]. Ligand binding induces transcriptional activation of PPAR*γ* that is followed by degradation [27]. This study identifies the E3 ubiquitin ligase E6-AP, as regulator of PPAR activity. The transcriptional activity of all three PPAR isotypes was inhibited by E6-AP. No changes were observed in ligand-induced transcriptional activity in the presence of E6-AP. PPAR*α* and E6-AP interacted in mammalian two hybrid assays, and by using an E6-AP mutant defective in ubiquitin ligase activity, we demonstrate that inhibition of PPAR*α* activity required the E3 ubiquitin ligase function of E6-AP. Interestingly, the presence of proteasome inhibitor MG132 had no effect on inhibition of PPAR*α* transactivation, suggesting that the proteasomal degradation is not required for E6-AP-mediated regulation of receptor transcriptional activity. This finding points to nonproteolytic functions of ubiquitination in modulating PPAR*α* activity. The multifaceted roles of ubiquitin in regulating protein localization, recruiting coregulators, and modifying chromatin structure are now well-recognized [7, 8, 28]. It would be of interest to examine these possibilities in regulation of PPAR*α* function by E6-AP.

The inhibition of PPAR*α* transcriptional activity by E6-AP is in contrast to previous findings with the progesterone receptor, where E6-AP coactivated receptor function, and the ubiquitin ligase activity was dispensable for its coactivating ability [11]. These observations suggest different mechanisms of E6-AP-mediated regulation of nuclear receptors. E6-AP is recruited to the ER-responsive pS2 promoter and is preferentially associated with E2-liganded ER*α* [29]. It would be of interest to determine if E6-AP is recruited to promoters of PPAR*α* target genes, as a mechanism of transcriptional regulation. Evidence also exists for NR-mediated transcriptional regulation of E3 ligases. Estrogen activation of ER*α* induces the expression of two ubiquitin ligases, MDM2 and Siah2 [20–22]. MDM2 is also regulated by the thyroid hormone receptor [30] orphan receptor TR3 [31], and constitutive androstane receptor (CAR) [32]. The breast cancer associated gene (BCA2) was identified as an E3 ubiquitin ligase and BCA2 expression correlates with positive ER status in breast tumors, suggesting that BCA2 and ER might be coregulated [21]. Our study shows that E6-AP mRNA and protein levels are increased in mice in response to PPAR*α* ligand clofibrate in wildtype but not PPAR*α* null mice, indicating a coordinate mode of regulation between PPAR*α* and E6-AP. In contrast to the ligand-independent decrease in PPAR*α* transcriptional activity mediated by E6AP in FaO cells, ligand treatment resulted in an increase in E6-AP expression in mice that was PPAR*α*-dependent. These results allude to the existence of a feedback loop between PPAR*α* and E6-AP wherein PPAR*α* increases the expression of a negative regulator for control of its transcriptional activity.

Studies in our laboratory have identified MDM2 as another ubiquitin ligase for PPAR*α* (unpublished results). MDM2 regulated the transcriptional activity of PPAR*α* by being recruited to the promoters of PPAR*α* target genes in response to ligand, and it interacted with the A/B domain of PPAR*α*. The various biological processes regulated by PPARs are crucial in control of disorders such as diabetes, inflammation, and cardiovascular ailments, and ubiquitin ligases such as E6-AP and MDM2 may present useful targets for pharmacological intervention and improved PPAR-based therapeutics.

In addition to contributing to understanding PPAR regulation by ubiquitination, other interesting connections can be made about the significance of the E6-AP-PPAR*α* interaction. E6-AP mediates ubiquitination and degradation of the Hepatitis C virus (HCV) core protein, which plays a crucial role in HCV-related liver disease [33]. HCV infections are associated with reduced hepatic PPAR*α* expression [34–37], and PPAR*α* is implicated in HCV core protein-mediated hepatic steatosis and dysregulated lipid metabolism [37]. The regulation of PPAR*α* by E6-AP may provide a basis for HCV-induced progression of liver disease, and is worthy of investigation.

## 5. CONCLUSIONS

This study identifies E6-AP, an E3 ubiquitin ligase, as a PPAR*α*-interacting protein that inhibited ligand-independent PPAR*α* transactivation and decreased the basal mRNA levels of PPAR*α* target genes. The E3 ubiquitin ligase function of E6AP was required for inhibition of PPAR*α* transcriptional activity, and this inhibition occurred in a proteasome-independent manner. E6-AP was induced in vivo in response to PPAR*α* ligand, and was regulated in a PPAR*α*-dependent manner. A better understanding of the role of E6-AP and other ubiquitin ligases in the regulation of PPARs could help improve treatment strategies against metabolic diseases.
